# Climate Change Perception and Mental Health. Results from a Systematic Review of the Literature

**DOI:** 10.3390/ejihpe14010014

**Published:** 2024-01-12

**Authors:** Vincenza Gianfredi, Francesco Mazziotta, Giovanna Clerici, Elisa Astorri, Francesco Oliani, Martina Cappellina, Alessandro Catalini, Bernardo Maria Dell’Osso, Fabrizio Ernesto Pregliasco, Silvana Castaldi, Beatrice Benatti

**Affiliations:** 1 Department of Biomedical Sciences for Health, University of Milan, Via Pascal, 36, 20133 Milan, Italy; francesco.mazziotta@unimi.it (F.M.); giovanna.clerici@unimi.it (G.C.); elisa.astorri@unimi.it (E.A.); francesco.oliani@unimi.it (F.O.); martina.cappellina@unimi.it (M.C.); fabrizio.pregliasco@unimi.it (F.E.P.); silvana.castaldi@unimi.it (S.C.); 2 Department of Biomedical Sciences and Public Health, Università Politecnica delle Marche, Via Tronto 10/a, 60100 Ancona, Italy; alecata@icloud.com; 3 “Aldo Ravelli” Center for Neurotechnology and Brain Therapeutic, University of Milan, 20157 Milan, Italy; bernardo.dellosso@unimi.it (B.M.D.);; 4 Department of Mental Health, Department of Biomedical and Clinical Sciences Luigi Sacco, University of Milan, 20157 Milan, Italy; 5 Fondazione IRCCS Ca’ Granda Ospedale Maggiore Policlinico, Via Francesco Sforza 35, 20122 Milan, Italy

**Keywords:** climate change, mental health, systematic review, depression, eco-anxiety, stress

## Abstract

Climate change is one of the main global challenges and influences various aspects of human health. Numerous studies have indeed demonstrated an association between extreme climate-related events and physical and mental health outcomes, but little is still known about the association between the perception/awareness of climate change and mental health. In accordance with the PRISMA 2020 guidelines, a search was conducted on PubMed and Scopus. The protocol was registered on PROSPERO. The included studies were original observational studies published in English, reporting the association between the perception/awareness of climate change and mental health. A total of 3018 articles were identified. A total of 10 observational studies were included. The period covered in the included studies ranged between 2012 and 2022. Climate change perception is consistently associated with adverse mental health effects across different types of estimates. In particular, the studies identified an association between a higher level of perception/awareness of climate change and depression, anxiety, eco-anxiety, stress, adjustment disorder, substance use, dysphoria, and even thoughts of suicide. Qualitative data underscore the impact on daily activities, contributing to feelings of loss and suicidal ideation. Moreover, climate change perception correlates with lower well-being and resilience. The association between awareness of climate change and mental health is a complex and still poorly explored phenomenon. The main limitations are the high heterogeneity in terms of exposure assessment and data reporting, which hinders quantitative analysis. These results show that climate change perception impacts mental health. Better understanding the phenomenon represents an opportunity to inform public health interventions that promote mental well-being.

## 1. Introduction

Climate change represents an urgent and paramount global challenge with profound implications for public health [1]. It exerts both direct and indirect impacts on various facets of individuals’ daily lives, making it a significant health threat [2]. One of the significant consequences of these interconnected issues is the increase in healthcare costs. For instance, in the United States, weather and climate events occurring between 2000 and 2009 increased healthcare costs by an estimated USD 819 million, resulting in more than 760,000 healthcare encounters and 1689 premature deaths [3]. The total health cost surpassed USD 15.5 billion [4].

The main driver of climate change is reported to be global warming, which, according to the Intergovernmental Panel on Climate Change Sixth Assessment Report (AR6) about climate change, is primarily due to human activities, the emissions of which have steadily increased in many regions since 1850 [5]. There are multiple ways in which climate change affects health; for example, rising temperatures can alter the epidemiology of various infectious diseases, including water-borne, food-borne, and vector-borne diseases [6]. This occurs due to changes in temperature, humidity, and pressure, which facilitate the proliferation of certain microorganisms and the survival of vectors like mosquitoes in latitudes different from their native regions [7]. Furthermore, climate change poses a risk to food safety and security, not only through food-borne diseases but also due to extreme weather events that reduce agricultural production, globally diminishing the availability of sufficient and nutritionally adequate food for the world’s population [8,9]. Extreme climate events can also directly impact the physical and mental health of individuals. When considering the impact of climate change on physical health, it is essential to note that between 2000 and 2016, the number of people exposed to heatwaves surged by approximately 125 million [10,11]. This increased exposure has resulted in increased hospitalization and death, primarily attributed to cardiovascular diseases, the incidence of which increased by 77.12% globally between 1990 and 2019 [12].

These are just some of the primary mechanisms through which climate change affects people’s health, without even considering the effects of desertification, which drives entire populations to migrate (climate migrations) [13], or the impact of climate change on air quality, with its related pollutants and allergens [14]. As mentioned, climate change also has effects on mental health [15]. A growing body of research links climate change to adverse mental health outcomes, including post-traumatic stress, depression (20–30% prevalence after extreme weather events), and anxiety following exposure to various climate events [16]. More in detail, recent research in the field has been primarily dedicated to the exploration of climate anxiety, eco-anxiety, climate grief, and the relatively new concept of solastalgia. These topics signify a burgeoning domain at the intersection of climate change and mental health. Solastalgia, for instance, pertains to the emotional distress stemming from the alteration, deterioration, and degradation of one’s surroundings, particularly in the context of the interplay between the environment, health, and place [17]. In a separate study, Knight delved into the intricate dimensions of climate anxiety and eco-anxiety and the correlation between anxiety and/or depression in the context of climate change [18]. Notably, it was observed that there is a lack of epidemiological data to establish the prevalence of distress and anxiety in this context. Furthermore, a recent Gallup poll disclosed that 54% of individuals aged 18 to 34 years, 38% of individuals aged 35 to 54 years, and 44% of individuals aged 55 or older express significant concerns about climate change [19]. Moreover, a recent meta-analysis pooled data from 19 studies and found that a 1 °C increase in mean monthly temperature was associated with a 1.5% (95% CI 0.8–2.2, *p* < 0.001) increase in the incidence of suicide outcomes [20].

As highlighted so far, numerous studies have aimed to measure the impact of climate change on human health and to explain the complex mechanisms behind these phenomena. Therefore, although the direct and indirect health consequences of climate change are increasingly well documented, less is known about the association between climate change perception/awareness and mental health. From a public health perspective, understanding the association between climate change perception/awareness and mental health holds crucial implications, particularly because it allows for the formulation of comprehensive policy frameworks that integrate mental health into climate change policies and vice versa. Moreover, building awareness on this topic emphasizes the need for interdisciplinary collaboration. In light of this, we aimed to collate, critically evaluate, and summarize all of the available evidence to assess the association between climate change perception/awareness and mental health outcomes.

## 2. Materials and Methods

The present systematic literature review was conducted in adherence to the Cochrane collaboration guidelines [21], and the results were reported according to the Preferred Reporting Items for Systematic Reviews and Meta-Analyses 2020 (PRISMA-2020) [22]. The review protocol was developed in advance and registered on the international database of prospectively registered systematic reviews (PROSPERO; registration number: CRD 42023445365).

### 2.1. Literature Search

For the current systematic review, articles were obtained from two databases: PubMed/MEDLINE and Scopus. The search engine was conducted simultaneously on 4 August 2023 by two authors. The research question was: Does climate change perception/awareness impact mental health? Therefore, the search strategy was developed by browsing keywords related to climate change and mental health outcomes. The full search strategy for each database is reported in Supplementary Table S1. Additional reference lists of included studies were all manually checked to identify any potentially relevant articles not previously included. Moreover, experts in the field were also consulted to include any additional potentially relevant articles that may not have been identified through database searches or by screening reference lists.

### 2.2. Inclusion/Exclusion Criteria and Selection Process

Inclusion/exclusion criteria were developed based on the population (P), exposure (E), comparison (C), outcome (O), and study design (S) approach. Details are reported in Supplementary Table S2. Briefly, only original observational studies published in international, peer-reviewed journals assessing the association between the perception/awareness of climate change and any type of mental health outcome among humans (any age, any sex), written in English, with the full text available and without any time filter (or date parameters), were considered eligible.

All the retrieved articles were screened based on a two-step process. Firstly, only the title and abstract were assessed in order to identify potentially relevant articles. Subsequently, the studies were examined to ensure that they fulfilled all the inclusion/exclusion criteria based on full-text assessment. The whole selection process was conducted in duplicate and any disagreement between the two authors was solved through discussion. If disagreement persisted, a senior author was consulted.

### 2.3. Data Extraction and Quality Assessment

From the included studies, relevant data were extracted and reported in a standardized and pre-defined spreadsheet using Excel (Microsoft Excel^®^ for Microsoft 365 MSO, USA, 2019). Data extraction was conducted in duplicate; therefore, in order to increase consistency among the two authors, the spreadsheet was pre-piloted on 3 randomly selected studies. The extracted data included the author, year of publication, country where the study took place, study period, study design, number of participants, age and gender, main population characteristics, number of people lost (attrition rate), tool used to assess the climate change perception, whether it was validated, definition of climate change, type of mental health outcome, diagnostic tool used to measure mental health outcomes, maximally adjusted effect size measurements along with the corresponding 95% CI, variables used for the adjustment, whether any funds were received to conduct the original study, and any declared conflicts of interest. Lastly, for each included study, the methodological quality was determined using the Newcastle–Ottawa Scale [23] (and its adapted version for cross-sectional studies [24]), which is a star system assessing three main domains: the selection of the study groups, the comparability of the groups, and the ascertainment of either the exposure or the outcome of interest. Based on previously adopted cut-offs [25], the studies were considered of high quality if the NOS score was equal to or greater than 7 points (out of 9).

## 3. Results

### 3.1. Literature Search

A total of 3018 records were identified after searching in PubMed/MEDLINE (*n* = 1605) and Scopus (*n* = 1413). No additional articles were included based on reference screening and expert consultation.

**Figure 1:**
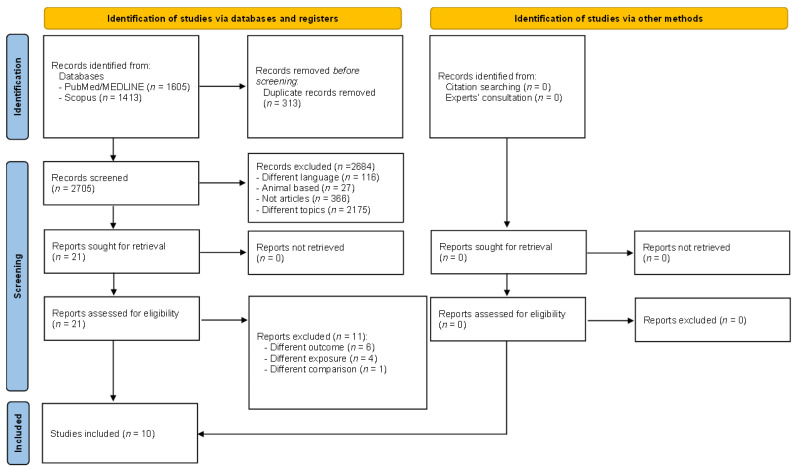
Flow diagram showing the selection process.

After preliminary exclusion of duplicates (*n* = 313), a total of 2705 records remained for assessment. After title/abstract screening, 116 records were removed because the language was not English, 27 records were removed because the studies were not performed on humans, 739 records were removed because they were not original papers (as for instance, reviews, book chapters, or letters to the editor), and 1809 records were removed because they focused on different topics, resulting in 14 records eligible for inclusion. After full-text evaluation, an additional four records were removed due to the following reasons: One record assessed the country’s vulnerability to climate change using the Notre Dame Global Adaptation Initiative Country Index [1] (different exposure), one record assessed the physical and psychological symptoms related to climate variations [2] (different outcome), one record assessed the experience of a climate change-related event() [3] (different exposure), and lastly, one record assessed the validity of a questionnaire related to the topic of the review without offering data on the association [4] (different aim). At the end of the selection process, a total of 10 records were included [5,6,7,8,9,10,11,12,13,14]. The selection process is depicted in Figure 1.

### 3.2. Main Characteristics of Included Studies

All of the included articles were cross-sectional studies conducted during 2012 [26] and 2022 [27]. Concerning the country where the studies took place, the United States of America was the most represented, in three studies [26,28,29] (one of which also included European undergraduate students [26]). Three studies were conducted in Europe (Norway [30], Germany [27], and Italy [31]), two studies were conducted in Australia [32,33], one study was conducted in Canada [34], and lastly, one study was conducted in Bangladesh [35]. The exposure of interest was generically defined as climate change perception in half of the included studies (*n* = 5) [27,32,33,34,35], whereas one study defined climate change perception as referring to environmental attitudes and pro-environmental behaviors [26]. Climate change preoccupation was assessed by two studies [30,31], whereas climate change awareness [29] and the emotional and functional impact on participants of climate change perceptions [28] were assessed by one study each. The majority of the included studies (*n* = 6) used a validated questionnaires to assess climate change perception/awareness [26,27,28,31,32,33]. At the same time, mental health outcomes were, in most cases, assessed by using validated and well-established tools, such as, for instance, the Patient Health Questionnaire (PHQ), the Generalized Anxiety Disorder (GAD) Scale, or the Depression Anxiety Stress Scales. Only three studies used tools (semi-structured interview or survey) developed ad hoc. Details are reported in Table 1.

### 3.3. Main Characteristics of Included Population

The population analyzed was mostly adults; only three studies include minors [30,33,34]. The majority was represented by young adult cohorts including high school, undergraduate, and graduate students. Only one study [34] included healthcare professionals and another primary care patients [29]. In all studies, there was a prevalence of the female sex among the respondents (average 58.66%). Only two studies reported data regarding attrition (referring to the loss of participants during the study, also including those without full data availability), which stood at 8% and 12% each [28,33]. The sample size was heterogeneous; the study with a smallest sample included 46 people [32], whereas the largest recruited 128,484 people [30].

### 3.4. Mental Health Outcomes

Several mental health outcomes were considered in the included studies, including depression (or depressive symptoms) [28,30,31,33], anxiety [28,29,31,32,33], eco-anxiety/ecological worries [26,32], stress [27,31,32,33], well-being [30,34], adjustment disorder [33], resilience [33], substance use [33], dysphoria [29], worry about the future [32], cognitive emotional impairment [28], cognitive functional impairment [28], feelings of loss [35], and feelings of suicide ideation [35]. In particular, one study assessed the prevalence of worry about the future, eco-anxiety, and stress and anxiety (combined) among those with higher climate change perception and found an occurrence of 93%, 89%, and 83%, respectively [32]. Despite different types of mental health outcomes and different types of estimates (odds ratio (the odds that an outcome will occur given a particular exposure), Pearson correlation (measures the strength and direction of the relationship between two variables), β coefficient (the estimated change in the dependent variable for a one-unit change in a predictor variable), or qualitative data)), all included studies found a significant association/correlation between climate change perception and mental health outcome. In particular, a higher perception of climate change was significantly associated with a higher risk of the most prevalent mental health disorders, such as major depression (β = 0.11 (±0.36) *p* > 0.05) [28], depressive symptoms (OR = 1.71 95% CI (1.10–1.42)) [30], anxiety (β = 0.37 (±0.34) *p* > 0.05) [28], or stress (β = 0.06, CI 95% (±0.03) *p* < 0.001) [27]. Moreover, the studies found a significant correlation between climate change perception and depression [29,31,33], anxiety in all age groups [29,33], or, specifically among young adults and adults [31], stress [31,33], adjustment disorder [33], substance use [33], dysphoria [29], and ecological worries [26]. Looking at the qualitative data, Kabir et al. reported that 75% of participants perceived that climate change impacted daily activities, which led to feelings of loss and increased feelings of suicidal ideation. Lastly, according to Middleton et al., climate change impacts environmental conditions, which in turn determines daily activities, indirectly affecting mental well-being. Well-being and resilience were also assessed in other two studies, which confirmed the trend [30,33]. Specifically, a higher perception of climate change was associated with a lower sense of well-being (OR = 0.91 95% CI (0.88–0.95)) [30] and less resilience (Pearson r = −0.14, *p* < 0.001) [33].

### 3.5. Quality Assessment of Included Studies

**Figure 2:**
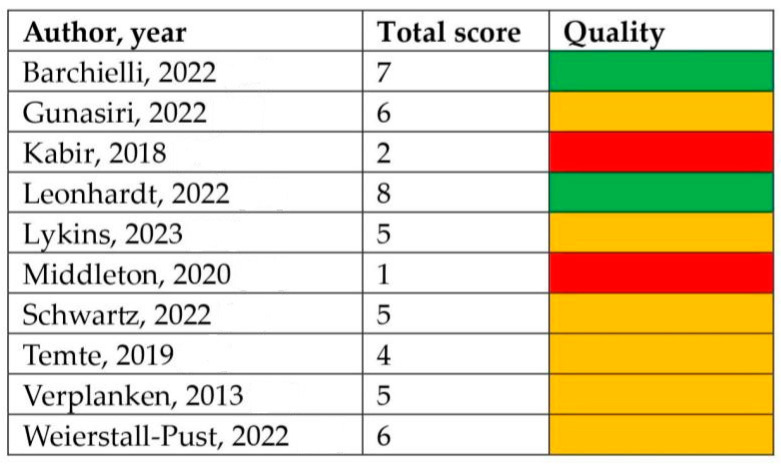
Quality assessment of the included studies. Green indicates high quality, orange indicates moderate quality, and red indicates low quality [26,27,28,29,30,31,32,33,34,35].

The results of the quality assessment, evaluated using the Newcastle–Ottawa Scale (NOS) adapted for cross-sectional studies [24], are reported in Figure 2. More than half of the studies (*n* = 6) were judged as moderate quality [30,31], two studies were considered of high quality, and the remaining two were considered of low quality [34,35]. Supplementary Table S3 reports the item-by-item quality assessment for each included study. Inter-rater reliability was assessed, and discrepancy among the two reviewers was around 5%. Disagreements were solved through discussion, and a final agreement was reached for all included studies. Additionally, information regarding adjustment (if any), conflict of interests, and funds was collected. In respect to this, only one study adjusted its statistical analysis for potential confounders [30]. In particular, sociodemographic characteristics, leisure activities, mental health, cannabis use, and alcohol intoxication were the selected variables. Two studies did not perform any type of adjustment [27,28]; in the remaining studies, adjustment was not applicable (because only prevalence was estimated, qualitative data were gathered, or only a Pearson correlation was performed). Regarding the declaration of conflicts of interest, all of the studies except for two reported no conflicts, with the remaining two studies not having reported this information [33,35]. Lastly, concerning funds received, only two studies did not receive funds [30,32].

## 4. Discussion

In the current systematic review, we aimed to compile, assess, and summarize all evidence exploring the relationship between climate change perception/awareness and various mental health outcomes. Indeed, grasping the link between climate change perception/awareness and mental health is pivotal because it helps to develop a holistic policy framework that intertwines mental health considerations with climate change policies and vice versa. Out of 3018 articles, at the end of the selection process 10 articles were included in the analysis. The main reason for exclusion was because the majority of the retrieved studies assessed the association between direct exposure to extreme weather events (or natural disasters) and mental health. On the contrary, in the current review, we only focused on the association between climate change perception/awareness and mental health outcomes. In fact, climate change is one of the main challenges of our society, considering its implication in terms of the environment, society, and health [36]. The results of our review show the complexity of the association between climate change perception and various mental health outcomes. A wide range of mental health outcomes have been associated with climate change perception. These include depression, anxiety, eco-anxiety, stress, adjustment disorder, substance use, dysphoria, and even thoughts of suicide. Despite the extensiveness of these mental health outcomes and the heterogeneity found both in terms of the mental health outcome measurements and estimation used among the studies, these results suggest a certain level of consistency, indicating that the statistically significant associations found are not just a random occurrence. Moreover, the strength of the associations is quite alarming but not entirely surprising considering the urgency of the climate crisis. In particular, major depressive disorder, depressive symptoms, anxiety, and stress were the most frequently studied mental health symptoms in association with climate change perception/awareness, and they also turned out to be the most prevalent mental health issues associated with climate change perception/awareness. For example, an increase in climate change perception/awareness was associated with an average increase of 1.71 in depressive symptoms [30]. Similarly, when increasing climate change perception/awareness, GAD-7 scores (measuring anxiety) increased by 0.4 units [28]. These data might, at least partially, explain the global mental health crisis [37], contributing to the widespread nature of so-called eco-anxiety, which is a relatively new term that reflects the growing sentiment of people who feel a sense of loss, helplessness, and frustration caused by their inability to cope with climate change [38]. Eco-anxiety adds a layer of complexity to the mental health landscape, highlighting the need for specialized support and interventions [39].

Furthermore, our results also highlighted the significance of the indirect effects of climate change perception/awareness. Specifically, qualitative data from Kabir et al. reveal the profound impact of climate change on daily life [35]. Participants reported that climate change-induced disruptions to their routines led to feelings of loss and intensified thoughts of suicide. Similarly, the findings of Middleton et al. underscore the intricate web of causation, where changes in the environment indirectly affect mental well-being [34]. This suggests that addressing climate change’s impact on mental health requires a holistic approach that encompasses both environmental and psychological dimensions [40]. Lastly, climate change perception seems to be also associated with lower levels of well-being and reduced resilience. This implies that individuals who are more attuned to the environmental crisis may find it more challenging to maintain a positive outlook and cope with adversity [41], suggesting that addressing climate change is an environmental concern and a public health urgency [42]. Consequently, if it is true that awareness of climate change has increased over the past decades, along with potential negative effects on mental health, as demonstrated by the results of the present review, it could be equally true that interventions aimed at developing climate change awareness might yield positive outcomes in terms of environmentally friendly behaviors and, hypothetically, manage the negative effects on mental health.

From this perspective, resilience might be framed in relation to ecological citizenship, which encompasses citizen participation in the moral and/or political aspects of dealing with global environmental problems. More in detail, compared to other forms of citizenship, which mainly refer to political participation in the decision-making process, ecological citizenship is primarily interested in behavioral changes and therefore in underlying attitudes [43]. From this perspective, regulation, education, and climate change-oriented incentives might have the opportunity to turn the negative effects of climate change perception/awareness in virtuous individuals into collective pro-environment attitudes and behaviors.

### 4.1. Potential Biological Mechanisms

Several factors, including socio-behavioral aspects, culture, information, and preparedness, influence how people experience and cope with climate change [44]. Empirical studies have begun to establish links between climate change and mental health consequences [16]. Climate change can impact mental health directly (e.g., heatwaves), indirectly in the short term (e.g., during extreme events like floods and hurricanes), or indirectly in the long term (e.g., through prolonged droughts, sea-level rise, deforestation, and forced migration) [45]. These events can trigger psychiatric conditions such as post-traumatic stress disorder, mood disorders like depression and anxiety, increased suicide rates, substance use, and aggressive behavior [46]. Moreover, vulnerable populations, including women, the elderly, children, individuals with pre-existing psychiatric conditions, those with low income or limited social networks, and indigenous/native communities, are most affected by climate change’s mental health consequences [45]. Nevertheless, the complexity arises from the diversity of factors being measured and the methods used to assess climate change’s effects make it difficult to firmly identify biological pathways and the cause–effect association between climate change and mental health [47]. Moreover, the impact of climate change on environmental changes contributes to climate change perception/awareness, altering lifestyle or driving environmentally motivated migration and thereby affecting the ability to cope with the stress derived thereof.

### 4.2. Limitations and Strengths

Systematic reviews of literature are considered to be at the highest level of evidence, but some aspects should be considered before generalizing the results. First, heterogeneity in terms of exposure assessment and definition, as well as in mental health outcome measurement, was detected. Second, due to differences in effect size estimation, it was not possible to perform a statistical pooling of the data retrieved, preventing the possibility of quantitively combining data. In fact, our results show an association between climate change perception/awareness and mental health outcomes, but the strength of this association and the exact relationship between these two phenomena remain unclear. Nevertheless, we offered an accurate evaluation of the available evidence. In fact, in the current review, we followed international guidelines for conducting and reporting systematic reviews, which helped us to exhaustively retrieve evidence on climate change perception/awareness and its impact on mental health outcomes. However, despite multiple databases being searched to retrieve all relevant articles, only 10 studies were retained at the end of the selection process. In fact, we only explored existing research published in peer-reviewed journals and restricted our search to articles published in the English language. This might have led to some selection bias, such as, for instance, governmental reports or health agency reports not having been taken into account. However, we are confident that the most relevant articles and data are commonly published in international peer-reviewed journals and that missing publications would be insignificant. Moreover, the transparency in inclusion/exclusion criteria application and their a priori definition contribute to minimizing selection bias. Furthermore, a risk of bias appraisal was conducted in order to assess the quality of the included studies, particularly to identify potential sources of bias derived from primary research. From this perspective, it can be stated that there was a noticeable shortage of high-quality literature on this topic, particularly regarding aspects related to statistical analysis. Additionally, it should be considered that reviews are based on published data and, therefore, are intrinsically prone to publication bias. In fact, it is highly risky to not publish some “negative” or not significant results, and consequently they are not assessed in reviews. However, the relatively low number of included studies might be due to the novelty of the topic instead of potential publication bias, considering that the first included study was published in the last decade. Despite climate change research being relatively consolidated, the assessment of its perception/awareness on mental health is new. In fact, the novelty of the current study is that, despite the already well-known association between the immediate impacts of climate change (or extreme weather events) and mental health, climate change might also indirectly negatively impact mental health. In fact, the psychological distress related to climate change perception might be linked to concerns about the future and the loss of the environment. Finally, our review included studies conducted both in developed and in developing countries, and therefore, the results might be able to be trans-culturally extended.

### 4.3. Implications for Public Health Policies and Future Research

The results of our systematic review are relevant in terms of supporting healthcare professionals and policymakers in making informed decisions about public health policies. In particular, the data from our review show the complexity and multifaceted aspects concerning the perception and awareness of climate change on mental health outcomes. Consequently, public health strategies should not only focus on the importance of educating about climate change, its implications in terms of environment, and how to prevent it but also consider the consequences for mental health outcomes. In fact, it should be considered that, according to the World Health Organization, the results obtained so far regarding global health, poverty reduction, and the contrast of health inequalities in terms of climate change have already been mined, particularly in terms of its effect on the implementation of universal health coverage. This is particularly true considering the impact of climate change on the burden of disease (both physical and mental health) and the worsening of health services access, especially during extreme weather events [36]. From this perspective, public health programs can be designed to provide psychological support, coping mechanisms, and resilience-building strategies for individuals experiencing distress related to climate change concerns. Moreover, this understanding has profound implications for the formulation of comprehensive policy frameworks. Integrating mental health considerations into climate change policies can enhance their effectiveness. Policymakers could develop strategies that not only mitigate environmental impact but also prioritize mental well-being. For instance, urban planning initiatives can incorporate green spaces and sustainable designs, promoting mental health in addition to addressing climate-related issues [48].

Simultaneously, mental health support systems should be equipped to help individuals cope with the anxieties and distress associated with climate change so that they can be aware of the issue and, consequently, have the tools to recognize the mental impact of climate change perception/awareness and know how to address it. More specifically, professionals should be able to promote emotional expression and open dialogue, whether for individuals or groups [44]. Additionally, they should work towards building self-confidence by nurturing effective approaches to coping with and adapting to challenges. Moreover, integrating climate change awareness and mental health considerations into public health policies is crucial to promoting the mental well-being of individuals, especially in a rapidly changing world. In fact, the awareness of the link between climate change perception and mental health emphasizes the need for interdisciplinary collaboration. Collaboration between mental health professionals, environmental scientists, and policymakers can lead to holistic approaches that address both the psychological and the environmental aspects.

Future research can benefit from the results outlined in the current review in several ways. Firstly, approximately half of the included studies did not use validated tools for climate change perception/awareness assessment. Therefore, it could be useful to develop and validate assessment tools that are able to screen for climate change perception/awareness, as well as conduct research to evaluate available assessment tools, making comparisons and highlighting similarities or differences. This could encourage future population-based studies with larger cohorts and more robust methods. Moreover, having valid tools that can be used (after cultural adaptation) in different countries could foster scientific collaborations and comparisons among different regions. Furthermore, future research should investigate the effectiveness of public health campaigns that aim to raising awareness about climate change and its implication on mental health, and research should focus on examining the integration of climate change considerations into mental health policies at both the national and the regional level. Moreover, considering the study design of the included studies (mainly cross-sectional studies with a predominantly quantitative approach), future studies could adopt a mixed method (both quantitative and qualitative) in order to better understand factors associated with both perception/awareness of climate change and mental health outcomes. Similarly, case-control and cohort studies could help strengthen the associations found in the cross-sectional approach, as well as help assess temporality. Additionally, cohort studies may be particularly relevant if conducted among young people, who are more exposed to the effects of climate change. In fact, deeply understanding the association of climate change perception/awareness among young people may represent an opportunity for public health policymakers who need to take action and implement policies for the short and medium–long terms. Lastly, experimental research (or, more specifically, quasi-experimental studies) could be used to better understand causality as it relates to climate change perception and mental health outcomes.

## 5. Conclusions

In conclusion, in the current study, we provide a systematic review of the literature on the perception/awareness of climate change on a broad range of mental health outcomes. Despite the heterogeneity of the retrieved studies, they were all concordant in detecting an association between the perception/awareness of climate change and negative mental health outcomes. These results highlight the importance of re-thinking the climate change issue—not only focusing on the environmental aspects or the direct association between catastrophic climatic events and physical and mental health outcomes but also considering the impact of climate change perception/awareness. In fact, the perception/awareness of climate change can lead to increased levels of stress, anxiety, depression, and a range of other mental health challenges.

## Figures and Tables

**Table 1:** Data extracted from the included studies.

Author, Year [Ref]	Country	Study Period	Study Design	Sample Size	Attrition	Population Characteristics	Climate Change Assessment	Validation	MH Outcome	Diagnostic Assessment	Effect Size (95% CI) *p*-Value
Barchielli, 2022 [31]	IT	n.a.	C-S	1831	n.a.	Access to electronic devices and internet connection; age 47.71; F 61%	4-item 5-point Likert scale of an ad hoc questionnaire	Yes	Dep, Anx, and stress	DASS-21	Climate change preoccupation was correlated with Dep and Anx in all age groups and with Anx among young adults and adults (Pearson ranged between 0.100 and 0.391, *p* < 0.01.)
Gunasiri, 2022 [32]	AU	13 July–3 August 2020	C-S	46	0	Young people living in Australia; age 18–24; sex n.s.	Survey developed ad hoc	Yes	Worry about the future, eco-anxiety, stress, and Anx	Survey developed ad hoc	Worry about the future 93%; eco anxiety 89%; stress and anxiety 83%
Kabir, 2018 [35]	BD	January 2015–October 2016	C-S	125	n.a.	High-variety background; age 10–70; F 75, M 50	Semi-structured interview	No	Feelings of loss and feelings of suicide ideation	Semi-structured interview	75% of participants stated that climate change impacted daily activities and led to feelings of loss, thereby increasing feelings of suicide ideation.
Leonhardt, 2022 [30]	NO	2021	C-S	128,484	11,357	Pupils; age 13–19; F: 60,959	Single 5-point Likert scale questionnaire	No	DepSym and well-being	DMI derived from the HSCCW	DepSym: OR 1.71 (1.10–1.42) well-being: OR 0.91 (0.88–0.95)
Lykins, 2023 [33]	AU	March 2020	C-S	746	n.a.	Adolescents, young adults; age 16–25; F: 584, M: 152, 10 others	ETSEAI	Yes	Dep, Anx, stress, adjustment disorder, substance use, resilience	DASS-21; ADNM8, UNCOPE, BRS	Dep (r = 0.15, *p* < 0.001); Anx (r = 0.11, *p* < 0.01); stress (r = 0.19, *p* < 0.001); adjustment disorder (r = 0.21, *p* < 0.001); substance use (r = 0.10, *p* > 0.05); resilience (r = −0.14, *p* < 0.001)
Middleton, 2020 [34]	CA	November 2012–May 2013	C-S	116	n.a.	96 community members + 20 health professionals; 68 F, 48 M	Semi-structured interview	No	Mental well-being	Semi-structured interview	Climate change impacts environmental conditions, which in turn determines daily activities, indirectly affecting mental well-being.
Schwartz, 2022 [28]	USA	October and December 2020	C-S	323	12% (284/323 provided completed data on all variables)	Students; age 18–35; F 78.9%; transgender, non-binary, or other 2.1%	3-item 5-point Likert scale for climate change experience and 13-item Climate Change Anxiety Scale	Yes	Cognitive and functional impairment in CCA, MDD, GAD	PHQ-8 for MDD, GAD-7	MDD β = 0.11 (0.36), *p* > 0.05; cognitive impairment β = 1.53 (0.76), *p* < 0.05; GAD β = 0.37 (0.34), *p* > 0.05; functional impairment β = 1.85 (0.72), *p* < 0.05
Temte, 2019 [29]	USA	2013	C-S	571	n.a.	Adult primary care patients; age 18–96; M 183, F 357	Climate change composite score	No	Dep, Anx, and dysphoria	PHQ-9, GAD-7, and a combination of PHQ-9 and GAD-7	Dep (Chi-square = 0.178, n.s.) and Anx (Chi-square = 0.441, n.s.), dysphoria: r = 0.345, *p* < 0.001)
Verplanken, 2013 [26]	USA and EU	June 2012–July 2012	C-S	132	n.a.	Students and non-students; age 26; 39 M, 78 F, 15 not declared	5-item 5-point Likert scale	Yes	Ecological worries, including global warming, pollution, extinction of species, resource depletion, and deforestation	PSWQ for item “pathological worry,” B5I for item “big five personality traits,” EAI for item “enviromental attitudes”	Habitual ecological worry and environmental attitudes (r = 0.47; *p* < 0.001), pro-environmental behavior (r = 0.37; *p* < 0.001)
Weierstall-Pust, 2022 [27]	DE	April–May 2022	C-S	3094	n.a.	Adults (≥ 18 years) with fluent German and access to internet; F = 1560 M = 1534	5 items 5-point Likert scale climate change stressors	Yes	Stress symptoms	SSQ-25	β = 0.06 (0.03) *p* < 0.001

ADNM8: Adjustment Disorder New Module 8; Anx: anxiety; AU: Australia; BD: Bangladesh; B5I: Big Five inventory; BRS: Brief Resilience Scale; CA: Canada; CCA: cognitive emotional impairment in climate change anxiety; C-S: cross-sectional; DASS-21: Depression Anxiety Stress Scales; DE: Germany; Dep: depression; DepSym: depressive symptoms; DMI: Depressive Mood Inventory; EAI: Environmental Attitude Inventory; ETSEAI: Environmental Threat Subscale of the Environmental Attitude Inventory; EU: Europe; F: female; GAD-7: generalized anxiety disorder; HSCCW: Hopkins Symptom Checklist and Cantril ladder for well-being; IT: Italy; M: male; MDD: major depressive disorder; NO: Norway; n.s.: not specified; PHQ: Patient Health Questionnaire; PSWQ: Penn State Worry Questionnaire; SSQ-25: subclinical stress questionnaire; UNCOPE: alcohol and substance abuse screener.

## Data Availability

No new data were created or analyzed in this study. Data sharing is not applicable to this article.

## Supplementary Materials

The following supporting information can be downloaded at: https://www.mdpi.com/article/10.3390/ejihpe14010014/s1, Table S1: Search strategy for each database; Table S2: Detailed inclusion/exclusion criteria, defined according to PECOS (population, exposure, comparison, outcome, study design); Table S3: Item-by-item quality assessment of each included study, reported in alphabetical order.
