# Supplementary material for: Climate Change Perception and Mental Health. Results from a Systematic Review of the Literature
Source: Eur J Investig Health Psychol Educ. 2024 Jan 12;14(1):215–29. doi: 10.3390/ejihpe14010014 (PMC10814599; doi:10.3390/ejihpe14010014)
Supplement: Supplementary file 1 [file ejihpe-14-00014-s001.zip › ejihpe-2733129-supplementary.pdf]

Supplementary Table S1: Search strategy, for each databased

Sets	Topics	Pubmed/MEDLINE
Set 1	Climate change	(("climat*"[Title/Abstract] AND "change*"[Title/Abstract]) OR "Climate Change"[MeSH Terms])
Set 2	Mental health	((("mental*"[Title/Abstract] OR "psychiatric*"[Title/Abstract] OR "behaviour*"[Title/Abstract] OR "behavior*"[Title/Abstract]) AND ("disorder*"[Title/Abstract] OR "illness*"[Title/Abstract] OR "disease*"[Title/Abstract] OR "diagnosis"[Title/Abstract])) OR "Mental Health"[MeSH Terms] OR "Mental Disorders"[MeSH Terms])
Set 3	Combine Set 1 AND Set 2	
**Sets**	**Topics**	**Scopus**
Set 1	Climate change	(((KEY (climat* AND change*)) OR (TITLE (climat* AND change*))) OR ((KEY (climate AND sensitivity)) OR (TITLE (climate AND sensitivity))) OR ((TITLE (climate AND variability)) OR (KEY (climate AND variability))))
Set 2	Mental health	((KEY (mental* OR psychiatr* OR behaviour* OR behavior*)) OR (TITLE (mental* OR psychiatr* OR behaviour* OR behavior*))) AND ((TITLE (disorder* OR illness* OR disease* OR diagnos*)) OR (KEY (disorder* OR illness* OR disease* OR diagnos*)))
Set 3	Combine Set 1 AND Set 2	

Supplementary Table S2: Detailed inclusion/exclusion criteria, defined according to PECOS (Population, exposure, comparison, outcome, study design).

**Search Strategy**	**Details**
**Inclusion criteria**	P: adolescents and adults (including elderly), female and male<br>E: perception/awareness of climate change<br>C: lowest perception/awareness of climate change<br>O: Any type of mental health outcome (self-reported or assessed by neurological tests, and/or medical records, and/or clinical visit/examination, and/or imaging, and/or blood tests)<br>S: original, observational study (including cross-sectional, case-control, or cohort both prospective and retrospective studies), published as peer-reviewed articles in international scientific journals
**Exclusion criteria**	P: children<br>E: experience of climate change events, seasonality variability, meteorological events, vulnerability to climate change, extreme weather events, natural disasters, climate change measured as meteorological parameter variations<br>C: not applicable<br>O: other health outcomes<br>S: not original (reviews with or without meta-analysis), not performed among humans, not observational (as for instance trials), not published as peer-reviewed articles in international scientific journals (book, book chapter, thesis), no full-text papers (abstract, conference paper, letter, commentary, note)
**Language**	English
**Time filter**	none

Supplementary Table S3. Item-by-item quality assessment of each included studies, reported in alphabetical order

	selection				comparability		outcome		NOS evaluation		Additional information		
**Author, year**	**Item 1**	**Item 2**	**Item 3**	**Item 4**	**Item 5a**	**Item 5b**	**Item 6**	**Item 7**	**Total**	**Quality**	**Adjustments**	**Conflict of interests**	**Funds**
Barchielli, 2022 [31]	*	*	*	*	-	-	**	*	7	high	not applicable	no	no
Gunasiri, 2022 [32]	*	-	*	*	-	-	**	*	6	moderate	not applicable	no	no
Kabir, 2018 [35]	*	-	-	-	-	-	-	*	2	low	not applicable	not declared	not declared
Leonhardt, 2022 [30]	*	-	*	*	*	*	**	*	8	high	sociodemographics, leisure activities, mental health, cannabis use and alcohol intoxication	no	no
Lykins, 2023 [33]	*	-	-	*	-	-	**	*	5	moderate	not applicable	not declared	yes
Middleton, 2020 [34]	*	-	-	-	-	-	-	-	1	low	not applicable	no	yes
Schwartz, 2022 [28]	*	-	-	*	-	-	**	*	5	moderate	no	no	yes
Temte, 2019 [29]	*	-	-	*	-	-	*	*	4	moderate	not applicable	no	yes
Verplanken, 2013 [26]	-	-	-	*	*	-	**	*	5	moderate	not applicable	no	yes
Weierstall-Pust, 2022 [27]	*	*	-	*	-	-	**	*	6	moderate	no	no	yes
